# Improved Visualization of the Internal Branch of the Superior Laryngeal Nerve during Pharyngeal Endoscopic Subepithelial Dissection with Use of Red Dichromatic Imaging

**DOI:** 10.1002/deo2.70460

**Published:** 2026-11-04

**Authors:** Yukihisa Fujinaga, Yoshiki Tamada, Yohei Yamaguchi, Kyohei Matsuura, Hiroki Kachi, Masafumi Oyama, Hitoshi Mori, Satoshi Iwai, Akira Mitoro, Hitoshi Yoshiji

**Affiliations:** ^1^ Department of Gastroenterology Nara Medical University Nara Japan; ^2^ Division of Endoscopy Nara Medical University Nara Japan

**Keywords:** endoscopic subepithelial dissection, internal branch of the superior laryngeal nerve, pharynx, red dichromatic imaging, white‐light imaging

## Abstract

Endoscopic subepithelial dissection is a minimally invasive treatment for superficial pharyngeal neoplastic lesions, requiring preservation of important neural structures such as the internal branch of the superior laryngeal nerve (ibSLN). We report a case in which red dichromatic imaging (RDI) appeared to improve the visualization of a linear structure presumed to be the ibSLN compared with white‐light imaging (WLI). A 57‐year‐old woman underwent endoscopic subepithelial dissection for a 5‐mm superficial lesion on the pharyngoepiglottic fold. Both WLI and RDI revealed a whitish linear structure beneath the lesion, which was presumed to represent the ibSLN based on its anatomical location and course. The presumed nerve structure appeared to be more clearly delineated with RDI than with WLI before mucosal incision and remained identifiable after submucosal injection and during dissection. This enhanced visualization may have helped guide the selection of the mucosal incision line and dissection plane. En bloc resection was achieved without apparent nerve injury. No dysphagia, hoarseness, or other adverse events occurred postoperatively. These findings suggest that RDI may serve as a useful adjunct for visualization of the structure presumed to represent the ibSLN during pharyngeal endoscopic subepithelial dissection and may support nerve‐preserving strategies.

**Abbreviations**
ESD: endoscopic submucosal dissection
ibSLN: internal branch of the superior laryngeal nerve
RDI: red dichromatic imaging
WLI: white‐light imaging.

## 1 Introduction

Superficial pharyngeal cancers have been increasingly detected at an early stage through widespread image‐enhancing endoscopy. Techniques for removing these tumors may be minimally invasive and may preserve laryngeal and swallowing function. Endoscopic submucosal dissection (ESD) has become widely adopted for superficial pharyngeal cancer because it enables preservation of organ function and has demonstrated efficacy and safety [1].

Unlike gastrointestinal ESD, pharyngeal ESD presents unique anatomical challenges, including a narrow field, vascular and neural structures such as the internal branch of the superior laryngeal nerve (ibSLN) within the subepithelial layer, and underlying muscle or cartilage in some regions [2, 3]. Therefore, structures must be accurately identified intraoperatively, and dissection must be performed with caution to avoid structural injury, particularly nerve damage. Injury to nerves may cause dysphagia and hoarseness, which adversely affect postoperative quality of life [4, 5].

Red dichromatic imaging (RDI) is an image‐enhancing endoscopic technique in which long‐wavelength narrow‐band light is used to improve visualization of deep mucosal blood vessels and submucosal structures [6]. In gastrointestinal ESD, RDI enhances the visualization of bleeding points and blood vessels in the submucosal layer [7]. However, the utility of RDI in pharyngeal ESD, particularly with regard to nerve preservation, remains unclear.

We report a case of ESD for a superficial pharyngeal neoplastic lesion arising from the pharyngoepiglottic fold, in which RDI appeared to improve the visualization of a linear structure presumed to be the ibSLN and may have helped guide subepithelial dissection with an emphasis on nerve preservation. We also discuss the utility of RDI in pharyngeal ESD in the context of the existing literature.

## 2 Case Report

A 57‐year‐old woman had a history of endoscopic treatment for superficial squamous neoplasms, including oropharyngeal and multiple esophageal ESDs. Endoscopic examination revealed a 5‐mm lesion on the pharyngoepiglottic fold. Because ESD had already been planned for other pharyngeal lesions, diagnostic endoscopic subepithelial dissection was also performed for this small lesion at the recommendation of the otolaryngology team. The procedure was performed by otolaryngologists and endoscopists.

The EVIS X1 endoscopy system (Olympus, Tokyo, Japan) with GIF‐XZ1200 and GIF‐H290T endoscopes (Olympus) was used during the dissection. While the patient was under general anesthesia, we exposed the operative field using a Sato curved laryngoscope. After initial evaluation of the lesion with narrow‐band imaging (Figure 1a), WLI (Figure 1b) depicted a whitish linear structure beneath the lesion; because of its anatomical location and course, we assumed that it represented the ibSLN. RDI mode 1 depicted the presumed nerve more clearly as a white linear structure (Figure 1c). After local submucosal injection of hyaluronic acid, the nerve remained more clearly visible with RDI than with WLI despite adequate subepithelial elevation (Figure 2). After circumferential mucosal incision adjacent to the nerve, the nerve remained unclear with WLI but was clearer with RDI (Figure 3a,b). During the subsequent subepithelial dissection, RDI also delineated the boundary between the presumed nerve and the surrounding subepithelial tissue more clearly than did WLI (Figure 3c,d); this enhanced visualization may have helped guide identification of the dissection plane. A FlushKnife BTs 2.0 (DK2618JB20; Fujifilm Corporation, Tokyo, Japan) was used for dissection. Two milliliters of hyaluronic acid were injected into the submucosal layer. The lesion was successfully resected en bloc within 12 min, without bleeding or nerve injury (Figure 4). The procedure was performed by an endoscopist with experience in approximately 30 pharyngeal and 100 esophageal ESD procedures. Histopathological examination revealed high‐grade dysplasia with negative resection margins. During regular postoperative follow‐up, including laryngoscopic examinations, no adverse events, including dysphagia, hoarseness, or impairment of the swallowing or cough reflexes, were observed. No such abnormalities have been identified during the 10‐month follow‐up period to date.

**FIGURE 1:**
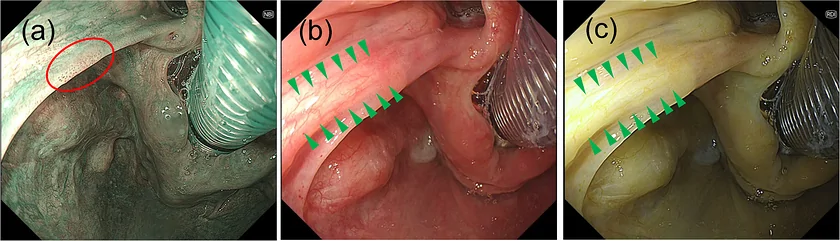
(a) Narrow‐band imaging depicted a 5‐mm brownish area on the pharyngoepiglottic fold (indicated by the red circle). (b) White‐light imaging depicted a whitish linear structure, which was presumed to be the internal branch of the superior laryngeal nerve based on its anatomical location and expected course. (c) Red dichromatic imaging demonstrated the nerve more clearly as a white linear structure.

**FIGURE 2:**
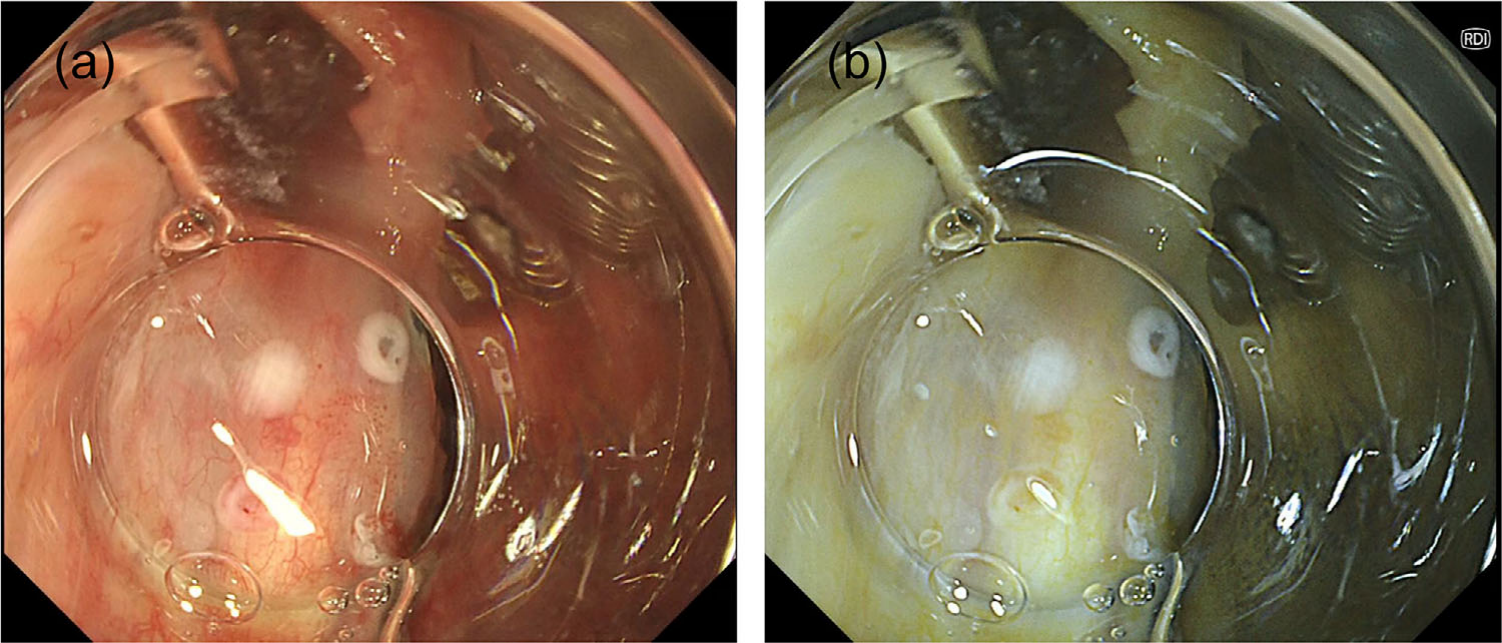
Comparison of white‐light imaging (WLI) (a) and red dichromatic imaging (RDI) (b) after submucosal injection of hyaluronic acid. Even after subepithelial elevation induced by the injection, the nerve appeared whiter and was more clearly visible with RDI than with WLI.

**FIGURE 3:**
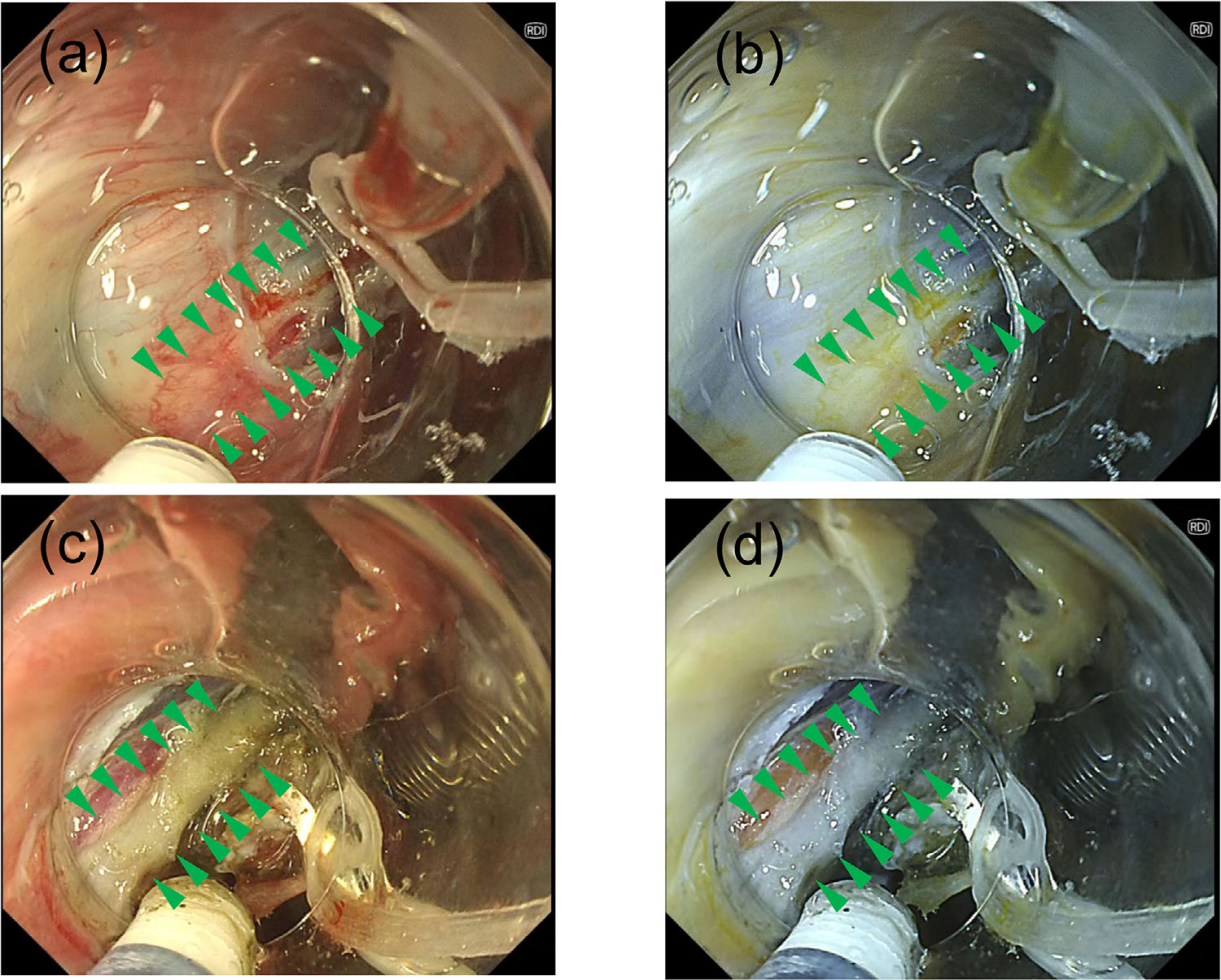
Comparison of white‐light imaging (WLI) (a) and red dichromatic imaging (RDI) (b) during endoscopic subepithelial dissection after circumferential mucosal incision. During subepithelial dissection, the nerve was less clearly visualized with WLI, but it was more clearly visualized with RDI. With RDI, the surrounding tissue appeared blue to gray due to the blue injection solution, providing greater contrast with the whitish nerve and facilitating identification of its course. The arrowheads indicate the nerve. The nerve was identifiable with both WLI (c) and RDI (d); however, the whitish appearance of the nerve was more prominent with RDI than with WLI.

**FIGURE 4:**
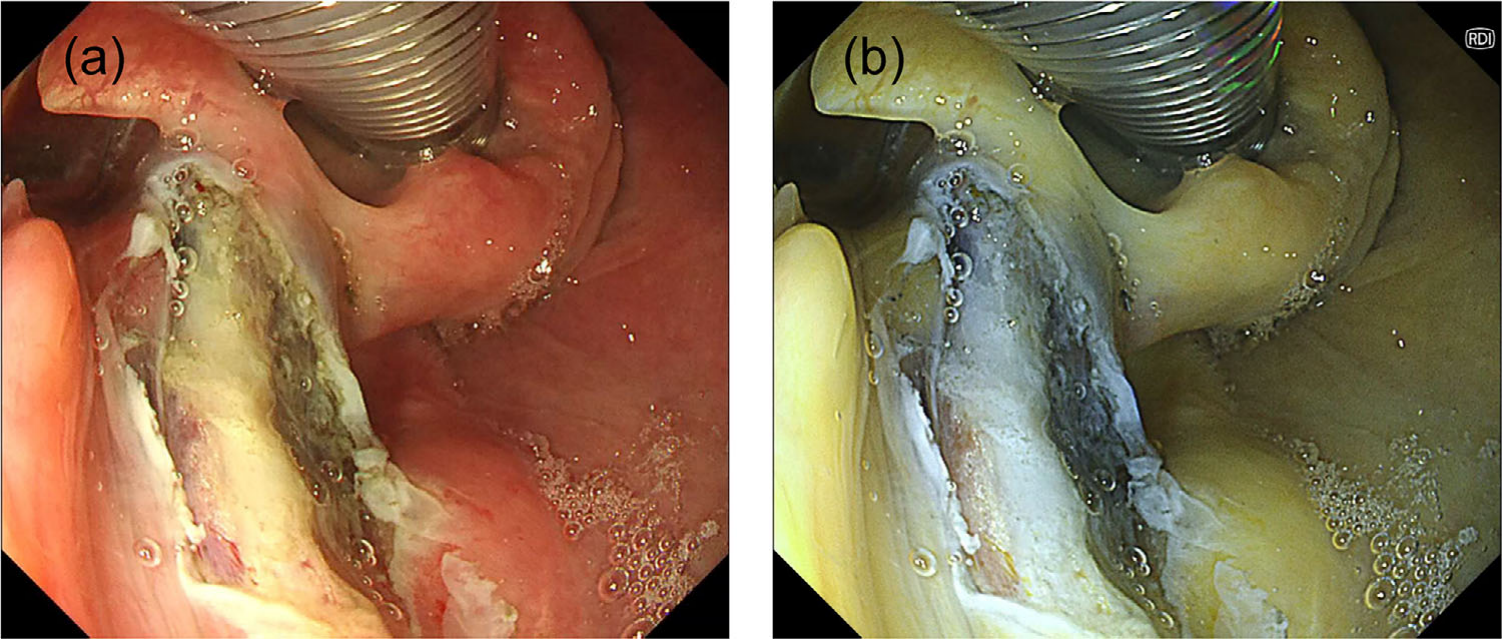
Endoscopic view after completion of endoscopic subepithelial dissection and en bloc resection with white‐light imaging (a) and red dichromatic imaging (b). The nerve was preserved without injury.

## 3 Discussion

RDI is an image‐enhancing endoscopy modality that is integrated into the EVIS X1 system. Its narrow‐band, long‐wavelength illumination consists primarily of 630‐nm red light and 600‐nm amber light, allowing greater tissue penetration than short‐wavelength light. It enhances the visualization of blood vessels in the submucosal and deep mucosal layers, facilitating the detection of bleeding points. This enhanced visualization helps clinicians identify the submucosal dissection plane during ESD, facilitates prompt endoscopic hemostasis, and may improve procedural safety and efficiency [6, 8]. RDI has also been utilized in other endoscopic settings. In the management of esophageal varices, RDI has been reported to facilitate endoscopic treatment by improving the visualization of deep veins and feeding vessels [9]. In ulcerative colitis, visualization of deep mucosal vessels through RDI has been investigated as a means of assessing inflammatory activity [9]. However, evidence regarding the utility of RDI during pharyngeal ESD remains limited, and its potential role in facilitating the identification of neural structures has not yet been adequately investigated.

The ibSLN, which arises from the vagus nerve, is the sensory branch of the superior laryngeal nerve. It passes through the thyrohyoid membrane and enters the larynx, where it divides into three branches. The superior branch courses toward the epiglottis, pharyngoepiglottic fold, and vallecula; the middle branch runs along the aryepiglottic fold; and the inferior branch courses toward the region of the false vocal fold, where it anastomoses with the peripheral branches of the recurrent laryngeal nerve [2, 3]. Based on this anatomical course, the linear structure observed in the present case was presumed to represent a branch of the ibSLN. Because this nerve plays a pivotal role in eliciting the swallowing and cough reflexes, injury during surgery may result in impaired swallowing reflexes as well as aspiration and dysphagia [4, 5]. Accordingly, during complete tumor resection by pharyngeal ESD, neural function must be preserved. Identification of the nerve under conventional WLI can be challenging; therefore, inadvertent intraoperative nerve injury is a potential risk.

In our patient, a whitish linear structure considered to represent the ibSLN was identifiable under conventional WLI. Under RDI, however, the structure was visualized more clearly as a distinct white linear structure, which may have facilitated its intraoperative identification. Importantly, the presumed nerve could be visualized before and after submucosal injection for subepithelial elevation, which may have helped guide the circumferential mucosal incision and subepithelial dissection while maintaining an appropriate dissection plane and avoiding apparent nerve injury.

Long‐wavelength light undergoes less tissue scattering and penetrates deeper into tissue than does short‐wavelength light. This optical property may explain why the ibSLN was identifiable with RDI before mucosal incision. Previous studies using label‐free diffuse reflectance spectroscopy (DRS) have reported high‐contrast nerve visualization at wavelengths of approximately 650 nm [10]. This observation suggests that the relatively long wavelength used in the present case may have contributed to the observed contrast. However, RDI was developed to enhance vascular contrast rather than to visualize nerves, and its imaging principle differs from that of DRS. Therefore, the mechanism responsible for the improved visualization observed in this case remains speculative, and further investigation is necessary.

In pharyngeal ESD, the risk of nerve injury is particularly high in areas such as the piriform sinus and aryepiglottic fold, where the ibSLN courses. Accordingly, early identification of the nerve, either before or in the early phase of the procedure, may help prevent inadvertent nerve injury by guiding the circumferential mucosal incision and the subepithelial dissection depth. RDI is therefore potentially useful for enhancing the visualization of neural structures and may help support nerve‐preserving strategies in pharyngeal ESD. An important limitation is that the apparent difference in the imaging findings between RDI and WLI was assessed without objective or quantitative evaluation. Further studies involving multiple cases and objective evaluations are warranted to establish the reproducibility and clinical utility of RDI for identifying neural structures during pharyngeal ESD.

In conclusion, this case suggests that RDI may improve visualization of the structure presumed to represent the ibSLN during pharyngeal ESD and may serve as a useful adjunct for identifying neural structures and supporting nerve preservation.

## Author Contributions

**Yukihisa Fujinaga**: conceptualization; visualization; writing – original draft; writing – review and editing. **Yoshiki Tamada**: data curation and investigation. **Yohei Yamaguchi**: data curation and investigation. **Kyohei Matsuura**: data curation and investigation. **Hiroki Kachi**: data curation and investigation. **Masafumi Oyama**: data curation and investigation. **Hitoshi Mori**: conceptualization and writing – review and editing. **Satoshi Iwai**: conceptualization and writing – review and editing. **Akira Mitoro**: writing – review and editing and project administration; supervision. **Hitoshi Yoshiji**: writing – review and editing and project administration; supervision. All authors drafted this article and revised it critically to provide important intellectual content. All authors approved the final version of the manuscript submitted for publication. All authors agreed to be accountable for all aspects of the work concerning accuracy and integrity.

## Funding

None. The authors have nothing to report.

## Conflicts of Interest

The authors declare no conflicts of interest.
